# Asthma in the Elderly

**DOI:** 10.1155/2009/858415

**Published:** 2009-10-27

**Authors:** Domenico Lorenzo Urso

**Affiliations:** Emergency Department, Vittorio Cosentino Hospital, 87062 Cariati, Italy

## Abstract

Bronchial asthma is a common problem with enormous medical and economics impacts. It is an inflammatory disease of the airways associated with intermittent episodes of bronchospasm. Asthma is not uncommon in the elderly patients. Prevalence of asthma is similar in older and younger adults. Asthma in the elderly patient is underdiagnosed because of false perceptions by both patient and physician. The high incidence of comorbid conditions in the elderly patient makes the diagnosis and management more difficult. Correct diagnosis is demonstrated with spirometry. The goals of asthma treatment are to achieve and maintain control of symptoms and to prevent development of irreversible airflow limitation. Asthma drugs are preferably inhaled because this route minimizes systemic absorption and, thus, improves the ratio of the therapeutic benefit to the potential side-effects in elderly patients.

## 1. Epidemiology

Asthma is an inflammatory disorder of the airway associated with airflow obstruction and bronchial hyperresponsiveness that varies in severity across the spectrum of the disease. The overall prevalence of asthma in adults and children varies between countries, with estimates of 7% in France and Germany, 11% in the USA, and 15%–18% in the United Kingdom [1]. Although asthma has been considered, for many years, a disease for childhood or young adulthood, its prevalence is similar in older and younger people [2]. An incidence study demonstrated rate of newly diagnosed asthma of 0.1% a year in those over 65 years of age [3]. Elderly asthmatic patients mainly include subjects who acquired the disease during childhood or adolescence and whose disease progressed over time or relapsed after periods of remission (Elderly asthmatic, long duration); however, the first manifestations of asthma may also occur in the late adulthood or after 65 years of age (elderly asthmatic, late onset) [4].

Little is known about the natural history of asthma in elderly patients, but there is evidence in literature that the elderly asthmatic patient is underdiagnosed [5, 6], undertreated [5, 6], has un higher risk of hospitalization, has a lower quality of life, and experiences greater morbidity and mortality [7]. Underdiagnosed and undertreated of asthma in the elderly may be due to diagnosis misclassification or under-reporting of symptoms [8]. Underestimation of the prevalence of asthma may be due to confusion with chronic obstructive pulmonary disease (COPD) [5].

The underdiagnosis may occur because of an age-related reduction in perception of shortness of breath [9]. In elderly patients there is a close relationship between the severity of wheezing complaints and impairment of the forced expiratory volume in 1 second (FEV1). Elderly patients with long-standing asthma have more severe airway obstruction than patients with recently acquired disease [4] but patients with newly diagnosed asthma experienced a more rapid rate of decline FEV1 than patients with chronic asthma [10]. Elderly patients did not show the elevated rate of allergy skin tests reactivity or high serum IgE levels [11]. However, elderly asthmatic patients have more evidence of atophy than age-matched controls without asthma as determined by increased immunoglobulin E (IgE) levels and positive skin test [12]. Inhaled corticosteroids (ICSs) have been show to slow this decline [13–15]. One can speculate, then, that if aggressive anti-inflammatory therapy had been started earlier in the course of the disease, some of this damage to the airways may have been prevented.

## 2. Definition and Assessment

Asthma is a chronic inflammatory disorder of the airways in which many cells and cellular elements play a role. The chronic inflammation is associated with airway hyperresponsiveness that leads to recurrent episodes of wheezing, breathlessness, chest tightness, and coughing, particularly at night or in the early morning. These episodes are usually associated with widespread, but variable, airflow obstruction within the lung that is often reversible spontaneously or with treatment [13]. Asthma is suggested by characteristic history of recurrent episodes of wheezing, breathlessness, chest tightness, and/or cough especially at night or in early morning. The certainty that asthma is correct diagnosis is increased when either clinic spirometry or pulmonary function tests demonstrate airflow obstruction that improves significantly, defined as both a 12% and 200 mL improvement in either FEV1 in response to inhaled bronchodilator [16]. However some studies indicate that an increase ≥ 10% of the predicted FEV1 after inhalation of a short-acting bronchodilator may be less subject to bias than measuring percent change from baseline and may have a higher likelihood of separating patients who have asthma from those who have COPD [17, 18]. Spirometry before and after using a bronchodilator should be an essential investigation although it may be difficult to perform in the elderly. In spite of all that in the SARA study Bellia et al. are obtained, in terms of acceptability and reproducibility of FEV1 in the vast majority of patients (aged 73 ± 6, 4 years) [19]. Measurement of airways responsiveness to methacholine in specialized pulmonary function laboratories may help to diagnose asthma [20].

Conventional assessment of asthma severity has combined assessment of symptoms, amounts of *β*-2 agonist used to treat symptoms, and lung function (Table 1). Asthma is classified as follows: Step 1: intermittent, with symptoms occurring less than once a week and patients remaining asymptomatic with normal peak expiratory flow (PEF) between attacks; Step 2: mild persistent, with symptoms occurring more than once a week, but with < 1 attack · day^−1^; Step 3: moderate persistent, with daily attacks affecting activity, and Step 4: severe persistent, with continuous limited physical activity [13]. Before treating elderly patients with asthma, some other factors should be considered. Elderly patients with suspect asthma require a rigorous and systematic approach to their diagnosis and treatment. The first step in the care of these patients is evaluation and testing directed at determining that asthma is the correct diagnosis. There are a number of reasons why asthma is underdiagnosed in elderly people (Table 2) [21]. It is critical to make a diagnosis of asthma and to exclude other airway diseases (Table 3). Comorbid conditions together with a less typical clinical pattern have been shown to significantly affect misdiagnosis. Indeed, patients with an erroneous diagnosis of COPD have shown a lower Barthel index [19].

COPD is usually easy to distinguish from asthma but sometimes the distinction from late asthma in older patients, particularly in cigarette smokers, is difficult and may be impossible. Both diseases are characterized by the presence of airflow obstruction but have a distinct pathogenesis and require unique treatment approaches. In general, the degree of reversibility to a bronchodilator has been used to determine whether a patient has COPD or asthma. The fixed airflow limitation of most patients can be defined as a post bronchodilator FEV1 of < 80% pred (in the presence of a reduced FEV1/FVC) after a 7-to-14 day course of oral corticosteroids (40 mg per day for adults and 2 mg/kg per day in children) [23, 24]. A negative response may indicate COPD, or rarely refractory asthma (corticosteroid-resistant) [22, 25, 26]. The distinction between asthma and COPD basic simply on spirometric parameters is difficult; therefore there is a need for more discriminatory tests such as lung volume and the assessment of CO Diffusing capacity (DLCO). Lung volumes (TLC, FRC, RV, RV/TLC%) are elevated [27]. Moreover there is a significant difference in DLCO values between patients with COPD and asthma [28]. The decreased DLCO may be directly related to the loss of alveolar-capillary surface area that is associated with emphysema [27].

Other risk factors that may contribute to a poor response to conventional therapy must be excluding (Table 4) [22]. Some comorbid conditions may complicate asthma care. A recent study showed that sinusitis, heartburn, COPD, Congestive Heart Failure, and smoking are significantly higher in the over 65-year age group [29]. A number of medications used for comorbid conditions may worsen asthma (Table 5) [21]. ASA and NSAIDs are commonly prescribed in the elderly and may cause late-onset asthma. On other hand medication used for asthma worsens comorbid conditions (Table 6) [21]. Oral and typical *β*-adrenergic blocking agents [28] and other antiarrhythmic agents, including verapamil [30], and others with acknowledged *β*-blocker potential can exacerbate or cause asthma in those who are predisposed to the disease [14, 31].

## 3. Treatment

The goals for successful management of asthma [13] are to

1. achieve and maintain control of symptoms,
2. prevent asthma exacerbations,
3. maintain pulmonary function as close to normal levels as possible,
4. maintain normal activity levels, including exercise,
5. avoid adverse effects from asthma medications,
6. prevent development of irreversible airflow limitation,
7. prevent asthma mortality.

Whenever possible, medications that might induce or aggravate asthma should be withdrawn. In other respects, the management of asthma in the elderly does not differ from that recommended for other age group, although particular care should be taken in the selection of and instruction in the use of inhaler devices [14]. Asthma drugs are preferably inhaled because this route minimizes systemic absorption and, thus, improves the ratio of the therapeutic benefit to the potential side-effects. Aerosolized medications that are used to treat asthma are available as pressurized metered-dose inhalers (MDIs), dry powder inhalers (DPIs), and nebulized or “wet” aerosol. Asthma medications should be used at the minimum dose and frequency required to maintain acceptable asthma control.

Medications used to treat asthma are generally divided into two main categories: relievers and controllers (Table 7). Relievers are best represented by the inhaled short-acting *β*2-agonist (SABA). Inhaled ipratropium bromide (IB) is less effective but is occasionally used as a reliver medication in patient intolerant of SABA. Controllers include anti-inflammatory medications, such as inhaled glucocorticosteroids (ICSs), leukotriene-receptor antagonists (LTRAs), long-acting inhaled *β*2-agonist (LABA), sodium cromoglycate, and sustained release theophylline [13–15]. Controller medications used daily on a long-term basis to achieve and maintain the goals of asthma treatment [13].

ICSs are the most effective agents in this category. Several studies are demonstrated that treatment with ICS for 1 month or more significantly reduces the pathological signs of airway inflammation in asthma and airway hyperresponsiveness continues to improve with prolonged treatment. Local adverse effects from ICS include oropharyngeal candidiasis, disphonia, and occasional coughing from upper airways irritation. The use of a spacer (holding chamber) improves drug delivery from an MDI. Spacers also reduce ICS deposition in the mouth and oropharynx.

LABAs, including formoterol and salmeterol, are bronchodilator medications with activity persist for at least 12 hours. They should be considered when standard introductory doses of CSI fail to achieve control of asthma before raising the dose of ICS. The principal side effects are dose dependent and are mediated via receptors on vascular smooth muscle (tachycardia and tachyarrhythmia), skeletal muscle (tremor, hypokalemia due to potassium entry into muscle cells), and cells involved in lipid and carbohydrate metabolism (increase in blood free fatty acid, insulin, glucose, and pyruvate) [32].

LTRAs are a relatively new class of drugs for the treatment of asthma. There is a substantial body of evidence for their benefit in the management of chronic asthma in both adults and children, and particularly in specific types of asthma such as exercise-induced and aspirin-sensitive asthma.

## 4. Conclusion

Asthma is an important healthcare problem worldwide. Although the prevalence of asthma in older people is similar to younger people, this disease is underdiagnosed and undertreated. Asthma in the elderly patient adversely affects quality of life and results in a higher hospitalization rate and mortality. Despite international guidelines recommending appropriate therapy for asthma, they are, in elderly patient, often poorly managed The high incidence of comorbid conditions makes the diagnosis and management more difficult. Despite the availability of effective antiasthma drugs, many old patients with asthma remain uncontrolled. Failure to achieve adequate control of asthma reveals a gap between what is known to be effective in asthma care and what might be achieved if optimal medication combined with management strategies could be implemented.

## Figures and Tables

**Table 1:** Classification of asthma severity by clinical features [13].

*Intermittent*
* *Symptoms less than once a week
* *Brief exacerbations
* *Nocturnal symptoms not more than twice a month
* *FEV1 or PEF > 80% predicted
* *PEF or FEV1 variability < 20%
*Mild Persistent*
* *Symptoms more than once a week but less than once a day
* *Exacerbations may affect activity and sleep
* *Nocturnal symptoms more than twice a month
* *FEV1 or PEF > 80% predicted
* *PEF or FEV1 variability < 20%–30%
*Moderate Persistent*
* *Symptoms daily
* *Exacerbations may affect activity and sleep
* *Nocturnal symptoms more than once a week
* *FEV1 or PEF 60%–80% predicted
* *PEF or FEV1 variability > 30%
*Severe Persistent*
* *Symptoms daily
* *Frequent exacerbations
* *Frequent nocturnal asthma symptoms
* *FEV1 or PEF < 60% predicted
* *PEF or FEV1 variability > 30%

**Table 2:** Underdiagnosis of asthma [21].

Dyspnea is caused by aging
Reduction in perception of dyspnea
Self-limitation of activities
Social isolation
Depression
Misconception that adult-onset asthma is rare
Comorbid conditions

**Table 3:** Diseases that mimic asthma.

Chronic obstructive pulmonary Disease
Congestive heart failure
Bronchiectasis
Upper airway obstruction
Aspiration or inhaled foreign body
Hyperventilation/panic disorder
Churg-Strauss syndrome and other vasculitides

**Table 4:** Risk factors [22].

Unidentified exacerbating factors
* *Unidentified allergens
* *Gastroesophageal reflux
Systemic diseases
* *Thyreotoxicosis
* *Carcinoid syndrome
* *Churg-Strauss syndrome and others vasculides
Drugs
* * *β*-blockers
* *Nonsteriodal anti-inflammatory drugs
Chronic infections
* *Mycoplasma
* *Chlamydia
Psycological factors

**Table 5:** Drugs that worsen asthma [21].

Hypertension	*β* blockers and ACE inhibitors

Glaucoma	Topical *β* blockers

Arthritis	Acetylsalicylic acid (ASA)/Non steroidal anti-inflammatory drugs (NSAIDs)

**Table 6:** Asthma drugs that worsen comorbid conditions [21].

*β* agonist	Arrythmias
	Tremors
	Hypertension
	Hypokalemia

Theophylline	Gastroesophageal reflux
	Tremors
	Insomnia

Corticosteroids	Existing osteoporosis

**Table 7:** Recommended medications by level of severity [13].

Level of severity	Daily controller medications	Other treatment options
Intermittent asthma	None necessary	

Mild persistent asthma	Low-dose ICS	Sustained-release theophylline LTRAs
		Sodium cromoglycate

Moderate persistent asthma	Low-to-medium dose ICS plus LABA	Sustained-release theophylline LTRAs
		Sodium cromoglycate

Severe persistent asthma	High-dose ICS plus LABA plus one or more of the following, if need Sustained-release theophylline LTRAs	
	Oral CS	
